# The screen inferiority depends on test format in reasoning and meta-reasoning tasks

**DOI:** 10.3389/fpsyg.2023.1067577

**Published:** 2023-03-09

**Authors:** Xun Wang, Luyao Chen, Xinyue Liu, Cai Wang, Zhenxin Zhang, Qun Ye

**Affiliations:** ^1^School of Psychology, Zhejiang Normal University, Jinhua, Zhejiang, China; ^2^Key Laboratory of Intelligent Education Technology and Application of Zhejiang Province, Zhejiang Normal University, Jinhua, Zhejiang, China

**Keywords:** reasoning, meta-reasoning, screen inferiority, medium, test format

## Abstract

Influential work has confirmed screen inferiority in reading tasks that reading on screen is less productive than reading on paper. Recent researches suggest that poor cognitive performance in screen environments may be primarily due to cognitive defects rather than technological flaws. Although some studies have explored screen inferiority in reasoning tasks from cognitive and metacognitive perspectives, related theories have yet to be enriched. Here, we found that screen inferiority exists in reasoning performance regardless of the test format (multiple-choice VS. open-ended), which may result from shallow processing consistent with the previous findings. However, meta-reasoning monitoring showed screen inferiority only in the multiple-choice test format. Our results indicate that the screens exhibit robust inferiority in reasoning scores, while the influence of the media on meta-reasoning may vary with external triggers. Our research may shed light on how to conduct efficient reasoning in the screen age.

## Introduction

In today’s education, online learning is becoming an increasingly popular mode. Millions of students worldwide have switched to online courses during the current COVID-19 pandemic. However, previous studies have shown that, compared to paper-based learning, learners tend to perform worse and have poorly calibrated self-assessments of their knowledge when learning on screen (Ackerman and Lauterman, 2012; Singer and Alexander, 2017; Kong et al., 2018; Clinton, 2019; Ronconi et al., 2022). This difference is often defined as screen inferiority.

Screen inferiority has been found in a large number of studies in recent years that have examined the effects of the media on reading comprehension and meta-comprehension. Specific phenomena include lower test scores, poorer calibrated metacognitive monitoring, and less effective effort regulation (Ackerman and Goldsmith, 2011; Ackerman and Lauterman, 2012; Lauterman and Ackerman, 2014). Although early researchers suggested that technological flaws contributed to this inferiority, recent studies have found that cognitive causes are more likely (Mizrachi, 2015; Jian, 2022; Lizunovaa et al., 2022). Specifically, it is the electronic devices that provide contextual cues that lead people to shallower processing, resulting in inferior cognitive performance.

However, screen inferiority is not always observed. Ackerman and Goldsmith (2011) chose participants with a strong paper preference to study the effect of time frame on screen inferiority. No significant differences between media were found under a limited time frame. In addition, Ackerman and Lauterman (2012) also replicated this experiment, selecting participants with a low preference for paper, and found that screen inferiority was only found when the time limit was known in advance and not when participants were unexpectedly interrupted after the same amount of study time.

Notably, in studies that have used reading comprehension tasks to examine the effects of the media on metacognition, texts often take up an entire page or even several pages. Such long texts often cause confusion about technical disadvantages and in-depth processing. To address this issue, Sidi et al. (2017) employed short problem-solving tasks (6 logical questions with a success rate of less than 20%) to examine whether screen inferiority remains when the reading burden is minimized. These tasks are briefly phrased to minimize reading burden and substantially reduce technology disadvantage while preserving required cognitive effort. Sidi et al. replicated Ackerman and Lauterman's (2012) time frame procedure using long texts, selecting a high cognitive population (SAT scores in the top 20%) as participants. This experiment found that solvers in the screen group had lower success rates under time pressure and were significantly overconfident. In addition, one remarkable finding of this experiment was the superiority of working on the screen under the ample time condition in terms of success and efficiency. This finding undoubtedly has important insights. However, the finding is limited to one condition in their Experiment 1.

The above studies suggest that the existence of screen inferiority is conditional; for example, the effects of the media change with the time frame. However, more conditions remain to be investigated. In previous studies, the task material was mostly misleading cognitive conflict problems or challenging logical problems. So, does the screen inferiority still exist for simple, non-misleading reasoning tasks? The current study will test the effects of the media on reasoning problems of varying difficulty, using conflict and non-conflict cognitive questions. Moreover, realistic educational needs were taken into account in the selection of reasoning tasks with different levels of difficulty. Given that learning to solve problems is an integral part of the curriculum in many school subjects, especially STEM (science, technology, engineering, and mathematics; van Gog et al., 2020), whether online reasoning learning differs from traditional paper-based reasoning learning is a question worth exploring. A meta-analysis (Clinton, 2019) found that the inferential reading tasks showed the same screen inferiority as the literal reading tasks, although inferential reading tasks were considered more complex than literal reading tasks (Basaraba et al., 2013). Therefore, we hypothesized that performance on both conflict and non-conflict questions would be worse on the screen than on paper.

In addition, the screen inferiority was reduced under certain conditions, especially when directing participants to recruit more intensive mental effort to the task than they would engage spontaneously. For example, Lauterman and Ackerman (2014) replicated the screen inferiority found by Ackerman and Lauterman (2012) under time pressure. They then demonstrated two easily applicable methods for overcoming screen inferiority: gaining experience with the difficult learning task and a requirement to generate keywords summarizing the essence of the text after a delay. This study suggests that simple task characteristics that encourage in-depth processing may help reduce screen inferiority.

Task characteristics are one level of heuristic cues, which are information and beliefs about factors affecting performance in a task as a whole (Ackerman, 2019). Examples include test type (open-ended vs. a multiple-choice test format), time frame (pressure vs. loose), and environment (e.g., computer vs. paper; indoor vs. outdoor). Assuming that the presence of screen inferiority disregards question type, the current study will examine other task characteristics that may reduce screen inferiority. Given that learners often take different types of chose to manipulate test type as a task characteristic that would be helpful in educational practice. Furthermore, for critical thinking questions, a multiple-choice test format (MCtf) was used to ease challenges compared to an open-ended test format (OEtf; Stanovich, 2009). In other words, the OEtf requires more cognitive effort than the MCtf. Therefore, we hypothesize that the OEtf will reduce the screen inferiority.

### The present study

To test our hypotheses, we evaluated the effects of the media, screen versus paper, and conflict versus non-conflict questions on performing problem-solving tasks. Conflict questions use the Cognitive Reflection Test (CRT; Frederick, 2005). This type of task contains both cognitive and metacognitive challenges. Such problems are designed so that the first solution that usually comes to mind is a wrong but predictable one. We constructe non-conflict version in which the first intuition was the correct answer (see Experiment 1, Materials section for problem descriptions; see Appendix for specific problems).

To delve into the metacognitive processes involved, we used the meta-reasoning framework (Ackerman and Thompson, 2015). Ackerman & Thompson proposed a meta-reasoning model based on meta-memory research and dual processing theories (DPT) and defined meta-reasoning as the monitoring and control of reasoning tasks and problem-solving. The meta-reasoning process is divided into two sub-processes: meta-reasoning monitoring and meta-reasoning control. Meta-reasoning monitoring collects relevant clues to reasoning and integrates them into judgments that assess any sense of rightness and falseness, after which meta-reasoning control evaluates these right and wrong perceptions against thresholds to determine subsequent responses (Ackerman and Thompson, 2017).

Considering the intuitive misleading nature of the CRT and meta-reasoning monitoring, we employed the two-response paradigm, a common paradigm for meta-reasoning studies, in the current study. The paradigm requires participants to respond twice to each question in a series of reasoning questions. First, participants are required to provide a quick intuition-based answer to the reasoning question. After participants have given their initial answer, the question is presented again with sufficient time to reconsider it and give a final answer. After the participants have provided each response, they are required to report their metacognitive judgment of the answer. The judgment after providing the initial answer is the feeling of rightness (FOR), whereas the judgment after giving the final answer is the final confidence judgment (FJC; Thompson et al., 2011; Ackerman and Thompson, 2015, 2017).

The two-response paradigm was chosen for this experiment for several reasons. First, the paradigm allows us to observe differences in the performance of the same subject at different stages of reasoning processing. Because in the time-limited initial response phase, participants were encouraged to engage in intuitive, shallower processing, while in the unlimited final response phase, participants were encouraged to engage in analytical, deeper processing. Second, the paradigm can capture the performance of participants’ meta-reasoning judgments at different stages of reasoning processing. In the current experiment, we collected data for both FOR and FJC. Finally, the paradigm allowed us to observe, to some extent, the effect of the time frame on the same participant. Specifically, the initial response was time-limited, in which participants were under previously known time pressure. The final response was completely unlimited, which can be considered as a completely relaxed state with no time pressure.

In summary, we used the two-response paradigm in Experiment 1 to examine reasoning and meta-reasoning performance on different problem-solving tasks of varying difficulty on screen and paper. As previously stated, if the existence of screen inferiority was found in Experiment 1, the current study will test whether the task characteristic (test format) reduces screen inferiority. Therefore, Experiment 2 replicated Experiment 1 but changed the test format intending to diminish the screen inferiority by encouraging in-depth processing.

## Experiment 1

In Experiment I, we aimed to explore the effects of media on reasoning and meta-reasoning monitoring. Since working in computerized environments is associated with shallower cognitive processing that leads to inferior cognitive performance (Sidi et al., 2016), participants may show screen inferiority in reasoning tasks. In addition, studies have demonstrated that screen-related contextual cues are associated with inferior metacognitive processes (Ackerman and Goldsmith, 2011), suggesting that participants’ meta-reasoning judgments may be unreliable on screen. We hypothesized that the participants might show impaired performance in the success rate and the meta-reasoning on the screen.

### Methods

#### Participants

Thirty-eight individuals were recruited from the local university and received monetary compensation for participating in this experiment. Four participants were excluded as they did not answer the questions as required, resulting in a sample of *N* = 34 (26 females; *M*_age_ = 20, *SD*_age_ = 1.40). We determined the sample size based on a power analysis that indicated 30 subjects would be sufficient to detect a medium-sized effect (d = 0.25) with 90% power. All participants had normal or corrected visual acuity and were computer literate. All participants provided written informed consent approved by the ethics committee of Zhejiang Normal University.

### Materials

Reasoning task materials were adapted from Ackerman and Zalmanov (2012). They include 16 conflict and 16 non-conflict questions, averaging 35 characters per question. The conflict questions refer to the problems in which the intuition and heuristic thinking induced by the topic information (i.e., the intuitive initial response) conflicts with the actual logic, probability, mathematics, or other rules. In conflict questions, people are prone to false or misleading answers. For example, the famous bat and ball problem (Frederick, 2005). “A bat and a ball cost $1.10 in total. The bat costs $1 more than the ball. How much does the ball cost?” Most people’s initial answer is $0.1, but upon reflection, the correct answer is $0.05 (Kahneman, 2011). The corresponding heuristic responses were consistent with the correct logical-mathematical responses in the non-conflict questions. For example, a non-conflict version of the ball and racket problem: “A bat and a ball cost $1.10 in total. The bat costs $1. How much is the ball?” For each question, a four-alternative multiple-choice test format version was constructed using one correct option (for example, the above question is $0.05), one misleading distractor (for example, the heuristic wrong answer is $0.1), one common distractor ($0.5), and “no correct answer.” The “no correct answer” option was set up to reduce participants’ guessing.

To determine the difficulty of the questions, 20 college students were recruited online for a pre-experiment. They completed 16 conflict questions and 16 non-conflict questions without time pressure. The correct rate of conflict questions was about 68%, and the correct rate of non-conflict questions was about 87%, showing that the question types are discriminative. These questions were divided into two versions equally, each consisting of 8 conflict questions and corresponding 8 non-conflict questions. The comprehensive difficulty and question type of each part were similar, and there was no significant difference in the number of words in these questions, which were 560 Chinese characters and 551 Chinese characters, respectively.

### Procedure

To determine the initial response time, 28 participants were recruited for a reading test. The procedure was adapted from Bago and De Neys (2017), in which participants read reasoning questions and options and then quickly picked an option at random. Different types of questions require additional reading times, and there is no precise time limit for the initial response in the dual-response paradigm (Kruglanski, 2013). Therefore, using the “average reading time” criterion to determine the time limit for the initial reaction is a practical solution. The average reading time of the participants was 6.77 s per question (*SD* = 2.38 s), and we took the nearest integer reading time, 7 s, as the initial response time limit.

**Figure 1:**
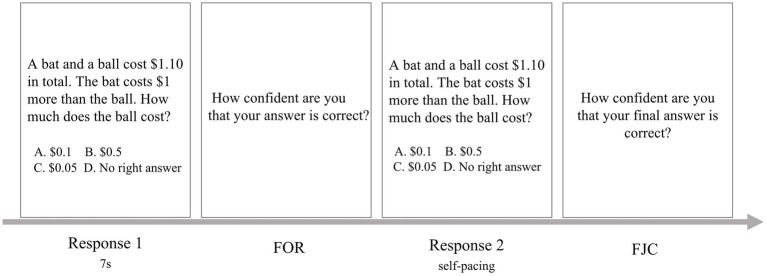
Example of the single-trial procedure in Experiment 1.

Given the nature of the experiment, participants were tested individually in a quiet laboratory space. Half of the participants answered 16 questions (8 conflict; 8 non-conflict) on a computer screen and then 16 on paper, and the other half was the opposite. Questions in each version and the order of the versions were randomly counterbalanced across participants. Before starting the formal experiment, the participants practiced 2 questions unrelated to the formal experiment to familiarize themselves with the procedure. Each experimental session lasted approximately 30 min. The experiment was administered *via* Psychopy (Peirce, 2007) and consisted of an initial and final responses (Figure 1).

In the initial response, each item was presented individually, displayed in the center of a computer screen or on paper. Participants were asked to come up with an initial answer that immediately came to mind in 7 s based on intuition. After responding, a new screen or a new piece of paper appeared where participants made FOR judgment by typing in the appropriate number, ranging from 0 (certain I’m wrong) to 10 (certain I’m right), on the keyboard or writing on the paper.

In the final response, each item was presented the same way as in the initial test, except that there was no time limit for the participants to answer the questions. Participants answered each item, then made FJC after their response using the same scale as FOR.

In terms of time control, the screen group was controlled by the Psychopy program, while the paper group was timed by the experimenter. In the timed phrase, participants in the paper group started the timer by pressing the button before beginning to respond and stopped responding after the timer expired. During the unlimited phrase, participants were not required to operate the timer. The timer had been fixed for 7 s, and participants only had to press the start button. The experimenter was present for both screen and paper groups to supervise the whole process.

### Results

#### Success rate

**Figure 2:**
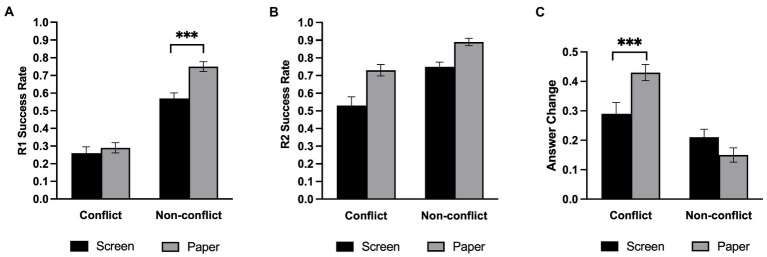
Success rate in Experiment 1. **(A)** The initial (R1) success rate. **(B)** The final (R2) success rate. **(C)** The probability of changing the initial answer. Error bars represent the standard error of the mean. ***p < 0.001.

Table 1 shows the average proportion of correct answers under each experimental condition. We conducted a 2 (Medium: screen, paper) × 2 (Question type: conflict, non-conflict) repeated-measures ANOVA on the proportion of correctly answered initial questions and showed the results in Figure 2A. The analysis revealed that the main effect of the medium was significant, *F*(1,33) = 15.96, *p* < 0.001, *η2 p* = 0.326, such that the paper group significantly better answered the questions than by the screen group. In addition, the main effect of question type was significant, *F*(1,33) = 152.50, *p* < 0.001, *η2 p* = 0.822, such that the success rate of the non-conflict questions is significantly higher than that of the conflict questions. Furthermore, there was a significant interaction between the medium and the question type, *F*(1,33) = 4.90, *p* = 0.034, *η2 p* = 0.129. Follow-up simple effects analysis revealed that the screen group reasoning performance was significantly lower than the paper group in the non-conflict questions, *F*(1,33) = 25.18, *p* < 0.001, *η2 p* = 0.433, while there was no significant difference between the screen group and paper group in the conflict questions, *F*(1,33) = 0.471, *p* = 0.497, *η2 p* = 0.014.

**Table 1:** Descriptive statistics (*M* ± *SD*) in Experiment 1.

	Conflict question		Non-conflict question	
	Screen	Paper	Screen	Paper
R1 Success Rate	0.26 ± 0.21	0.29 ± 0.17	0.57 ± 0.18	0.75 ± 0.16
R2 Success Rate	0.53 ± 0.29	0.73 ± 0.19	0.75 ± 0.15	0.89 ± 0.12
FOR	0.62 ± 0.13	0.52 ± 0.14	0.66 ± 0.14	0.69 ± 0.14
FJC	0.89 ± 0.12	0.84 ± 0.12	0.91 ± 0.10	0.93 ± 0.08
FOR Accuracy	0.36 ± 0.27	0.23 ± 0.19	0.09 ± 0.17	−0.06 ± 0.20
FJC Accuracy	0.36 ± 0.28	0.11 ± 0.14	0.16 ± 0.18	0.04 ± 0.13
Answer Change	0.29 ± 0.22	0.43 ± 0.16	0.21 ± 0.16	0.15 ± 0.14

All values are converted to a percentage. R1, initial response, R2, final response, FOR, feeling of rightness, and FJC, final judgement of confidence.

For the proportion of the final correctly answered questions (Figure 2B), a repeated-measures ANOVA showed that the main effect of the medium was significant, *F*(1,33) = 34.38, *p* < 0.001, *η2 p* = 0.510, questions were also significantly better answered on paper than on screen. As expected, the main effect of question type was also significant, *F*(1,33) = 28.77, *p* < 0.001, *η2 p* = 0.466. However, there was no significant interaction between the medium and the question type, *F*(1,33) = 1.26, *p* = 0.27, *η2 p* = 0.037.

Considering the null effect, we computed Bayes factors (BFs), which measure the strength of evidence for a given hypothesis (either the null or the alternative) based on both *a priori* hypothesis and the observed data (see Wagenmakers et al., 2018a,b for the benefits of using a Bayesian approach). Here, we report BF_01_ for any observed null effects, which provides a measure of the null hypothesis’s strength. Wagenmakers et al. (2018a) compiled guidelines for interpreting Bayes Factors adjusted from Jeffreys (1961) and reported that BFs of 1–3 indicate anecdotal evidence for the hypothesis, BFs of 3–10 indicate moderate evidence, and BFs >10 indicate strong evidence for the given hypothesis. BFs were calculated in JASP. The BF_01_ for the two-way interaction was 2.79, indicating weak support for the null hypothesis. This means that the data were 2.79 times more likely if the null hypothesis was true than if the alternative hypothesis was true. In subsequent analyses, BF_01_ will be reported for null effects.

#### Answer change

Participants were considered to have changed their answers if their final answers did not match their initial answers. The answer change rate is the number of questions with revised answers divided by the total number of questions. We conducted a repeated-measures ANOVA on the change in answer (Figure 2C) and found a significant effect on the question type, *F*(1,33) = 46.69, *p* < 0.001, *η2 p* = 0.586, and a non-significant effect on medium, *F*(1,33) = 1.85, *p* = 0.183, *η2 p* = 0.0535, BF_01_ = 2.62. Medium significantly interacted with question type, *F*(1,33) = 13.09, *p* = 0.001, *η2 p* = 0.284. Follow-up simple effects analysis revealed that there was no significant difference between the screen group and the paper group in the non-conflict questions, *F* (1,33) = 3.77, *p* = 0.061, but the paper group changed more questions than the screen group in the conflict questions, *F* (1,33) = 9.58, *p* = 0.004, *η2 p* = 0.225.

#### Meta-reasoning monitoring

We used the FOR and FJC to measure meta-reasoning monitoring in the current study. In the meta-reasoning framework, FOR is the prediction of the correctness of the initial answer, and FJC is the judgment of the possibility that the final answer is correct. The higher the FOR is, the more confident the participants are in the initial answer and the lower the possibility of changing answers. The original data (0–10) were converted by percentages (0–100%), and the descriptive statistical results are shown in Table 1.

**Figure 3:**
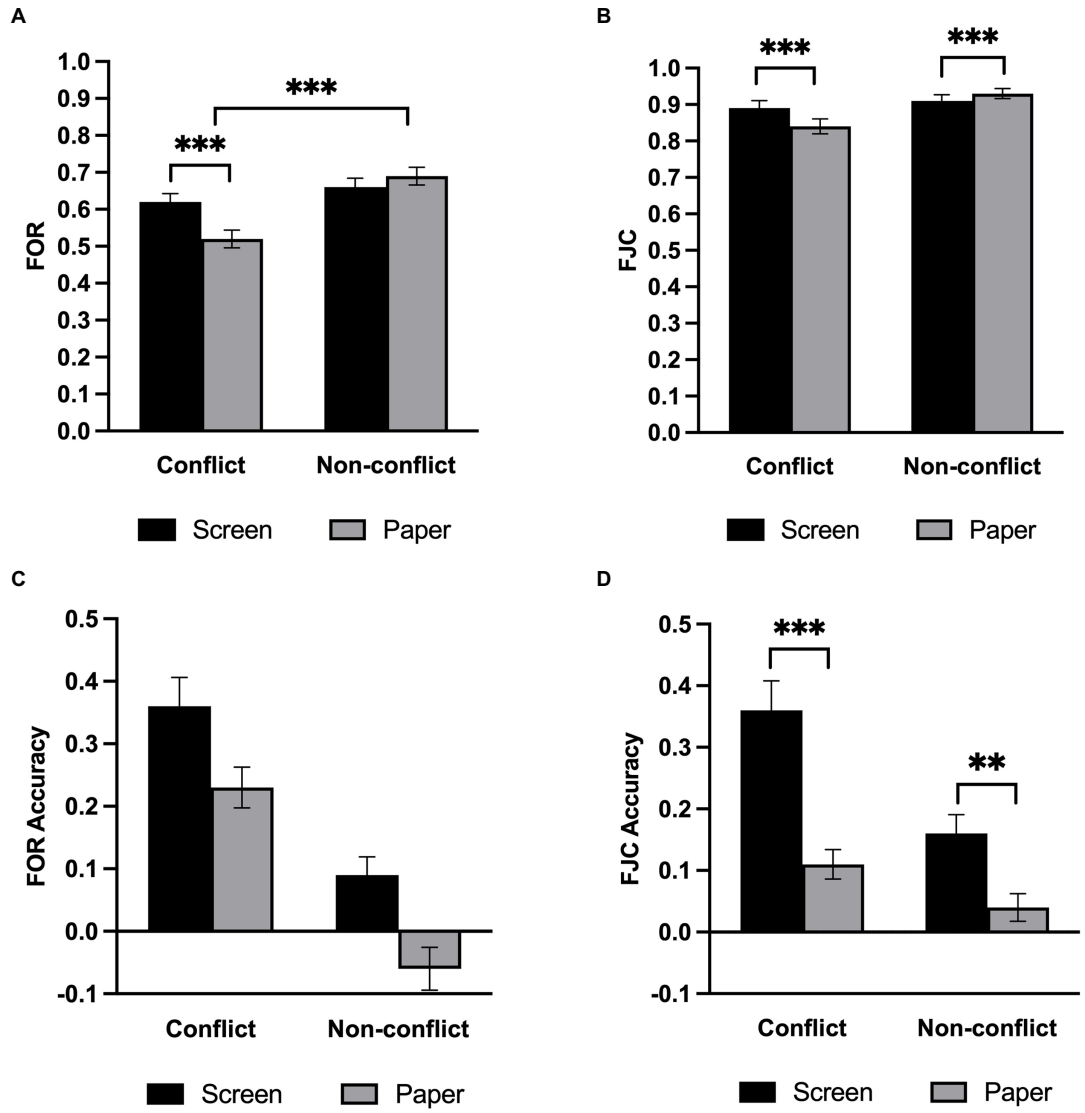
Meta-reasoning performance in Experiment 1. **(A)** FOR. **(B)** FOR accuracy. **(C)** FJC. **(D)** FJC accuracy. Error bars represent the standard error of the mean. **p < 0.01. ***p < 0.001.

We conducted a repeated-measures ANOVA on FORs (Figure 3A). The results revealed that the main effect of question type was significant, *F*(1,33) = 59.32, *p* < 0.001, *η2 p* = 0.643, such that the participants felt that the non-conflict questions were more likely to be correct than conflict questions. The main effect of the medium was not significant, *F*(1,33) = 2.80, *p* = 0.103, *η2 p* = 0.078, BF_01_ = 1.45. There was a significant interaction between the medium and the question type, *F*(1,33) = 21.03, *p* < 0.001, *η2 p* = 0.389. Follow-up simple effects analysis revealed that there was no significant difference between the screen group and the paper group in the non-conflict questions, but FOR on screen was significantly higher than that on paper in the conflict questions, *F*(1,33) = 12.44, *p* = 0.001.

We conducted a repeated-measures ANOVA on FJCs (Figure 3B). Results showed that participants gave higher confidence ratings to the non-conflict questions than to the conflict questions, *F*(1,33) = 29.88, *p* < 0.001, *η2 p* = 0.475, but showed no difference between the screen and the paper, *F*(1,33) = 1.50, *p* = 0.229, *η2 p* = 0.043, BF_01_ = 2.93. The two-way interaction was significant, *F*(1,33) = 12.71, *p* = 0.001, *η2 p* = 0.278, and follow-up simple effects analysis revealed that FJC on screen was significantly lower than that on paper in the non-conflict questions, *F*(1,33) = 5.33, *p* = 0.027, *η2 p* = 0.139, while the FJC on screen was significantly higher than that on paper in the conflict questions, *F*(1,33) = 7.74, *p* = 0.009, *η2 p* = 0.190.

#### Meta-reasoning monitoring accuracy

The current study used the absolute accuracy of overall performance prediction as an indicator of the meta-reasoning monitoring accuracy. Specifically, the absolute accuracy is computed by subtracting the actual accuracy rate from the judgment values (FOR - R1; FJC - R2) of the performance prediction. A positive value indicates overconfidence, while a negative value indicates underestimation. Furthermore, the closer the value is to 0, the more accurate the monitoring is.

We conducted a repeated-measures ANOVA on the FOR accuracy (Figure 3C). The analysis revealed that the main effect of the medium was significant, *F*(1,33) = 23.61, *p* < 0.001, *η2 p* = 0.417, in which the screen group showed a greater degree of overestimation. The main effect of question type was also significant, *F*(1,33) = 72.30, *p* < 0.001, *η2 p* = 0.687. However, the interaction of medium and question type was not significant, *F*(1,33) = 0.07, *p* = 0.788, *η2 p* = 0.002, BF_01_ = 4.09. To assess the FOR accuracy, we performed one-sample t-tests under each condition with 0. It turns out that the FOR accuracy values are significantly greater than 0 (*ts* > 3, *p* < 0.004) for all conditions except when participants answer non-conflict questions on paper instead (*t* = −2.10, *p* = 0.043).

To analyze the FJC accuracy, we also conducted a repeated-measures ANOVA (Figure 3D). Like FOR, the main effect of medium, *F*(1,33) = 40.00, *p* < 0.001, *η2 p* = 0.548, and question type, *F*(1,33) = 21.12, *p* < 0.001, *η2 p* = 0.390, were both significant. Furthermore, there was a significant interaction between the medium and question type, *F*(1,33) = 6.39, *p* = 0.016, *η2 p* = 0.162. Follow-up simple effects analysis showed that participants were more overconfident of the conflict questions, *F*(1,33) = 36.71, *p* < 0.001, *η2 p* = 0.527, than of the non-conflicting questions, *F*(1,33) = 10.59, *p* = 0.003, *η2 p* = 0.243, although they were overconfident of both question types on screen. Notably, the overestimation degree of the conflict questions on screen was significantly higher than that of the other conditions, all *t*s > 4, *p* < 0.001.We also performed one-sample t-tests under each condition with 0 to assess FJC accuracy and found that the FJC accuracy values are significantly greater than 0 (*ts* > 4.6, *p* < 0.001) for all conditions except when participants answer non-conflict questions on paper (*t* = 1.7, *p* = 0.098).

### Discussion

In experiment 1, we found that the reasoning performance on screen was significantly lower than that on paper in both phases, regardless of question types, which is consistent with the findings of screen inferiority in the field of reading (e.g., Mangen et al., 2019).

In addition, the results for the FOR accuracy indicated that the participants experienced a greater degree of overestimation on the screen. The FJC accuracy analysis showed that participants’ overconfidence was significantly higher on screen than on paper. These results show that the participants’ metacognitive monitoring of their reasoning process on screen is inaccurate, which means that the meta-reasoning monitoring process also has screen inferiority.

## Experiment 2

In Experiment 1, a multiple-choice test format (MCtf) was used to test participants. Researchers may argue that MCtf cannot make full use of in-depth cognitive processing and higher-order thinking, and there are higher guessing components (Cronbach, 1988; Pennycook et al., 2015). However, solvers must generate their answers using a macro-level strategy when deciding how to construe the problem in an open-ended test format (OEtf; Ackerman and Zalmanov, 2012). In other words, OEtf encouraged solvers to put more cognitive effort into in-depth processing compared to MCtf. Besides, previous work has found that compared to MCtf (or recognition), OEtf (or free recall) can better utilize valid cues as metacognitive judgment clues (Kelley and Jacoby, 1996). To reduce screen inferiority, we replicated Experiment 1’s question type and medium manipulation but used an open-ended test format (OEtf), which requires in-depth cognitive processing, in Experiment 2. Considering that in-depth processing was associated with improved test scores and improved reliability of metacognitive monitoring in text learning (Thiede and Anderson, 2003; Lauterman and Ackerman, 2014), there would be no significant difference between screen and paper in reasoning performance and meta-reasoning monitoring accuracy. In other words, screen inferiority would not occur when a more cognitive effort is required.

### Methods

#### Participants

Participants were 35 local university students aged 18–25 (M = 20.4 years, SD = 1.42 years; 23 females) and received monetary compensation for their participation. All participants had normal or corrected visual acuity and were computer literate. All participants provided written informed consent approved by the ethics committee of Zhejiang Normal University.

#### Materials and procedure

The materials and procedure were identical to those used in Experiment 1 except that the test format was changed from MCtf to OEtf and scratch paper was allowed. Specifically, in Experiment 1, each question was presented with four options, while in Experiment 2, each question was presented with no options.

### Results

#### Success rate

**Figure 4:**
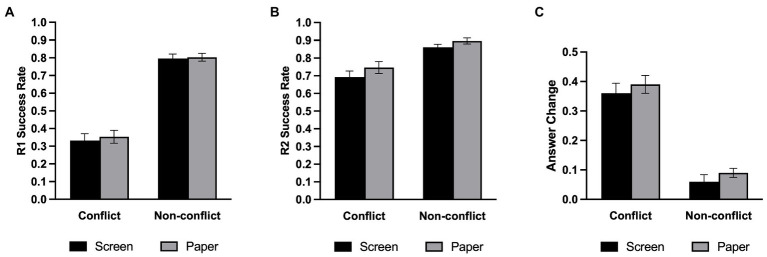
Success rate in Experiment 2. **(A)** The initial (R1) success rate. **(B)** The final (R2) success rate. **(C)** The probability of changing the initial answer. Error bars represent the standard error of the mean. ***p < 0.001.

Table 2 shows the average proportion of correct answers under each experimental condition. We conducted a 2 (Medium: screen, paper) × 2 (Question type: conflict, non-conflict) repeated-measures ANOVA on the proportion of initial correctly answered questions. As shown in Figure 4A, the main effect of question type was significant, *F*(1,34) = 167.74, *p* < 0.001, *η2 p* = 0.831, such that the success rate of the non-conflict questions was significantly higher than that of the conflict questions. There was no main effect of medium [*F*(1,34) =0.27, *p* = 0.610, *η2 p* = 0.008, BF_01_ = 3.53] nor a two-way interaction effect [*F*(1,34) =0.13, *p* = 0.721, *η2 p* = 0.004, BF_01_ = 3.84].

**Table 2:** Descriptive statistics (*M* ± *SD*) in Experiment 2.

	Conflict question		Non-conflict question	
	Screen	Paper	Screen	Paper
R1 Success Rate	0.33 ± 0.23	0.35 ± 0.22	0.80 ± 0.15	0.80 ± 0.13
R2 Success Rate	0.69 ± 0.20	0.75 ± 0.20	0.86 ± 0.10	0.90 ± 0.10
FOR	0.60 ± 0.16	0.60 ± 0.18	0.72 ± 0.15	0.74 ± 0.15
FJC	0.90 ± 0.08	0.92 ± 0.07	0.94 ± 0.06	0.95 ± 0.06
FOR Accuracy	0.27 ± 0.22	0.25 ± 0.21	−0.07 ± 0.18	−0.06 ± 0.20
FJC Accuracy	0.21 ± 0.19	0.20 ± 0.20	0.08 ± 0.11	0.05 ± 0.11
Answer Change	0.36 ± 0.20	0.39 ± 0.18	0.06 ± 0.14	0.09 ± 0.09

All values are converted to percentages. R1, initial response, R2, final response, FOR, feeling of rightness, and FJC, final judgement of confidence.

For the proportion of final correctly answered questions, we also conducted a repeated-measures ANOVA (Figure 4B). There was a significant main effect on the medium, *F* (1,34) = 7.30, *p* = 0.011, *η2 p* = 0.177, and participants performed significantly better on paper than on screen. The main effect of question type was significant, *F* (1,34) = 23.85, *p* < 0.001, *η2 p* = 0.412, and the success rate of the non-conflict questions were still significantly higher than that of the conflict questions. But there was no significant interaction between the medium and the question type, *F*(1,34) =0.32, *p* = 0.576, *η2 p* = 0.009, BF_01_ = 2.00.

#### Answer change

We conducted a repeated-measures ANOVA on the change in answer (Figure 4C) and found that the main effect of question type was significant, *F*(1,34) = 115.69, *p* < 0.001, *η2 p* = 0.77. However, the main effect of medium [*F*(1,34) = 0.352, *p* = 0.560, *η2 p* = 0.010, BF_01_ = 3.62] and the two-way interaction [*F*(1,34) = 1.02, *p* = 0.320, *η2 p* = 0.029, BF_01_ = 2.46] were not significant.

#### Meta-reasoning monitoring

**Figure 5:**
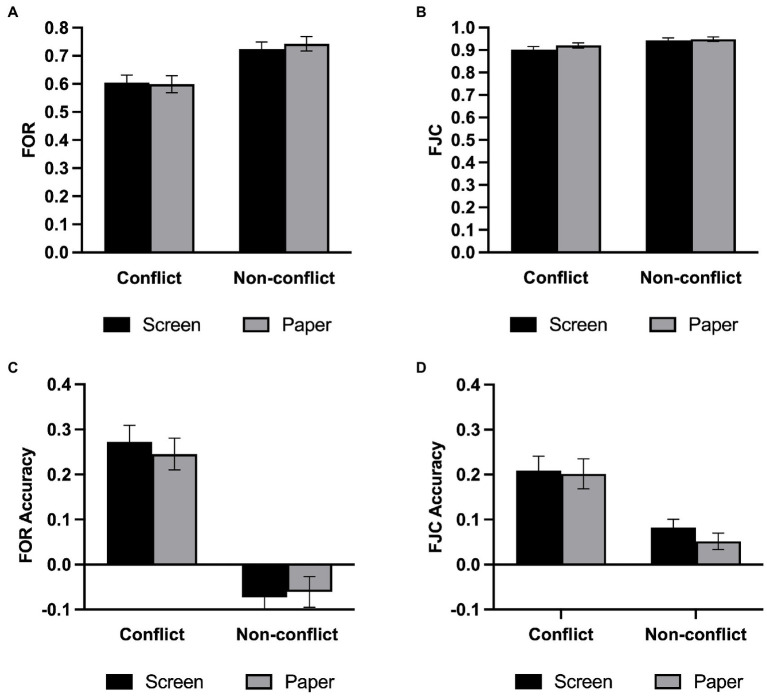
Meta-reasoning performance in Experiment 2. **(A)** FOR. **(B)** FOR accuracy. **(C)** FJC. **(D)** FJC accuracy. Error bars represent the standard error of the mean. ***p < 0.001.

We examined meta-reasoning monitoring in the same way as in Experiment 1, and the descriptive statistical results are shown in Table 2. As depicted in Figure 5A, a repeated-measures ANOVA on FORs revealed that the main effect of question type was significant, *F*(1,34) = 83.61, *p* < 0.001, *η2 p* = 0.711, such that participants felt that the non-conflict questions were more likely to be correct than the conflict questions. However, there was no main effect of medium [*F*(1,34) = 0.20, *p* = 0.657, *η2 p* = 0.006, BF_01_ = 3.95] nor a two-way interaction [*F*(1,34) = 0.71, *p* = 0.407, *η2 p* = 0.020, BF_01_ = 2.88].

We next examined FJC with a repeated-measures ANOVA on FJCs (Figure 5B) and found a significant main effect on the question type, *F*(1,34) = 16.60, *p* < 0.001, *η2 p* = 0.328, and participants were more confident in their responses to the non-conflict questions. There was no significant main effect on the medium, *F*(1,34) = 2.60, *p* = 0.116, *η2 p* = 0.071, BF_01_ = 1.49, and no significant interaction between the medium and question type, *F*(1,34) = 1.65, *p* = 0.208, *η2 p* = 0.046, BF_01_ = 1.92.

#### Meta-reasoning monitoring accuracy

Experiment 2 used the same indicator of the meta-reasoning monitoring accuracy as Experiment 1. We also conducted a repeated-measures ANOVA on the FOR accuracy (Figure 5C) and found that the main effect of question type was significant, *F*(1,34) = 82.43, *p* < 0.001, *η2 p* = 0.708. However, the analysis revealed that the main effect of the medium was not significant, *F*(1,34) = 0.09, *p* = 0.770, *η2 p* = 0.003, BF_01_ = 4.13, and the two-way interaction was not significant, *F*(1,34) = 0.87, *p* = 0.357, *η2 p* = 0.025, BF_01_ = 2.89. Then, we performed one-sample t-tests in the same manner as in Experiment 1. It turns out that the screen group was underestimated in the non-conflict questions, *t*(34) = −2.43, *p* = 0.021, and the FORs in the conflict questions were overestimated in both media, all *t*s > 5, *ps* < 0.001.

We conducted a repeated-measures ANOVA to assess the FJC accuracy (Figure 5D). Like FOR, the main effect of question type was significant, *F*(1,34) = 20.19, *p* < 0.001, *η2 p* = 0.373. Neither the main effect of medium [*F*(1,34) = 0.93, *p* = 0.342, *η2 p* = 0.027, BF_01_ = 3.10] nor two-way interaction [*F*(1,34) = 0.48, *p* = 0.495, *η2 p* = 0.014, BF_01_ = 3.62] was significant. We also performed one-sample t-tests and found that confidence judgment was overestimated under all conditions, all *t*s > 0, *p*s < 0.008.

### Discussion

Although there was no difference in the initial answers between the screen and the paper in experiment 2, participants’ final answer success rate on paper was better than on screen. In addition, the answer success rate of the non-conflict questions was higher than that of the conflict questions, regardless of the initial or final answer. One explanation for this finding is the attentional account that attention and comprehension affect people’s reasoning performance (Mata, 2020). Although forced to invest more cognitive effort in the OEtf, participants may not notice the traps (or detect the conflicts) in the conflict questions, leading to labor-in-vain.

However, in terms of the accuracy of meta-reasoning monitoring, consistent with our hypothesis, there was no difference between the screen and the paper. In other words, the screen inferiority of meta-reasoning monitoring may diminish by in-depth processing. Similarly, Sidi et al. (2017; Experiment 1) found no significant difference in overconfidence between the screen and the paper when solving problems without time pressure in OEtf.

## General discussion

Screen inferiority has been shown to exist in reading; specifically, electronic reading comprehension is poorer and less efficient than paper-based reading (Ackerman and Lauterman, 2012; Daniel and Woody, 2013; Lauterman and Ackerman, 2014; Delgado et al., 2018; Clinton, 2019). However, studies on how the media affects reasoning performance and meta-reasoning process are relatively rare. In the current study, we extended previous studies to reasoning tasks of varying difficulty and conducted two experiments that varied in test format to investigate the conditions under which screen inferiority occurs.

In Experiment 1, we compared participants’ answer performance and meta-reasoning accuracy on screen and on paper across different types of reasoning tasks. Our analysis showed that the participants’ success rate and meta-reasoning accuracy were inferior in the computerized environment regardless of question type, consistent with the hypothesis that screen inferiority exists in both reasoning performance and meta-reasoning process.

There are a few possible explanations for why the screen impaired reasoning performance and meta-reasoning accuracy. As previously indicated, research in the field of reading suggests that shallower processing on the screen may lead to a negative impact on reading performance (Ackerman and Lauterman, 2012; Lauterman and Ackerman, 2014; Sidi et al., 2016). This can be supported by the answer change rate. Changing one’s initial answer implies further deep thinking. In Experiment 1, the answer change rate of conflict questions on the screen was lower than that on paper, but there was no significant difference in the non-conflict questions. Generally speaking, conflict questions are misleading and require further processing through analytical thinking, while non-conflict questions are not misleading and can be answered correctly based on intuitive experience in a relatively short time. The lower answer change rate of conflict questions which required more cognitive effort provided evidence that people were performing shallower processing on the screen.

Another potential explanation is that inaccurate meta-reasoning judgments on the screen affected people’s subsequent reasoning performance. The relationship between metacognitive monitoring and cognitive processing depth is bidirectional. On the one hand, the reliability of metacognitive monitoring is related to the recruitment of cognitive effort (Thiede and Anderson, 2003; Lauterman and Ackerman, 2014); on the other hand, meta-reasoning monitoring is the core component of meta-reasoning that affects how people allocate cognitive resources and control subsequent reasoning (Dewey, 2022). Based on the feedback from the meta-reasoning monitoring process, the reasoner controls his or her object reasoning process, such as abandoning reasoning or changing the initial response. Previous studies have shown that when FOR is high, people tend to believe that the intuitive answer they gave the first time is correct without re-answering (Martin and Sloman, 2013; Ackerman and Thompson, 2017). It can be seen that FOR may play a role in regulating the level of effort in subsequent tasks, similar to Feeling of Knowing (FOK) and Judgement of Learning (JOL).

In Experiment 1, the analysis of FOR accuracy found a greater degree of overestimation on screen. It might be the overestimation that led to less in-depth processing. Participants were overconfident in their intuitive answers and tended to keep their initial answers, resulting in a lower success rate on screen. This was evidenced by the results of the FJC accuracy test, which showed that participants were significantly more overconfident on the screen than on paper. However, it should be noted that although there was no screen inferiority in the meta-reasoning monitoring accuracy in Experiment 2, the final answer performance on screen was lower than on paper. Therefore, the inaccuracy of metacognitive monitoring in the screen environment is the reason for the screen inferiority to some extent, but not decisive. More research is needed to determine the impact of meta-reasoning monitoring processes on people’s object-reasoning processes and how to improve reasoning performance through meta-reasoning processes.

As mentioned above, Experiment 2 did not completely eliminate screen inferiority for reasoning scores, although it did eliminate screen inferiority for meta-reasoning. Specifically, the analysis of Experiment 2 revealed that there was no significant difference in the initial answer success rate between the screen and the paper, but the final answer success rate on the screen was still inferior to that of the paper. Although we adopted the OEtf, which removed the distraction of misleading options, and provided scratch paper for participants to encourage in-depth processing. A possible explanation for this phenomenon is that attention and comprehension of the question were associated with reasoning performance (Mata, 2020). Delgado and Salmerón (2021) found that reading on a screen leads to distraction and more mind wandering when exploring the effects of reading medium and time frame on attention. They further argued that this unfocused reading leads, at least in part, to shallow reading and lower comprehension. People may not be able to sustain their attention on a screen for as long as they can on paper. In addition, the simple interview after the experiment showed that the participants had a preference for paper, which has been found in previous studies as well (Jeong and Gweon, 2021; Hargreaves et al., 2022). When they solved the questions on paper, they could concentrate more quickly and did not wander easily, whereas when they solved the questions on screen, they could not concentrate and could not respond to the answers immediately after reading the questions. This may also have contributed to the difference. Future research could further explore the role of attention in how the medium affects reasoning decisions.

From the time frame perspective, if the initial response phase is regarded as a time pressure condition, solvers in the screen group in Experiment 1 showed a lower success rate under time pressure, consistent with previous research (Ackerman and Lauterman, 2012; Sidi et al., 2017). In Experiment 2, We speculate that the lack of misleading/inspirational options in the OEtf resulted in no significant difference in success rates under the time pressure. The final response phase, in which we did not manipulate any time constraints, can be regarded as a loose time frame. However, we did not find that the screen group was superior in success rate, which differs from the conclusion of Sidi et al., 2017 (Experiment 1). This result should be treated with caution, as the present study was not an exact replication of Sidi et al., 2017.

Overall, as hypothesized, screen inferiority exists in the reasoning task regardless of question type and can be reduced by using simple task characteristics, which is well in line with previous research results (Lauterman and Ackerman, 2014). When asked to perform the screen-based MCtf, participants were more likely to allocate fewer cognitive resources to complete the task, resulting in poorer reasoning and meta-reasoning performance. However, when asked to complete the screen-based OEtf, which required more in-depth processing, participants focused their limited cognitive resources more on the task, and their reasoning and meta-reasoning performance did not show significant inferiority. Current research points in the direction of reducing screen inferiority. Returning to education, students are increasingly studying and testing online. To minimize the impact of screens on learners, instructors can add open-ended tests to their courses to increase students’ mental effort or add open-ended questions to online exams to improve students’ performance. It is worthwhile to further explore in future research how to sustain efficient learning in the screen age.

## Data availability statement

The raw data supporting the conclusions of this article will be made available by the authors, without undue reservation.

## Ethics statement

The studies involving human participants were reviewed and approved by Ethics Committee of Zhejiang Normal University. The patients/participants provided their written informed consent to participate in this study.

## Author contributions

CW, XW, and ZZ designed the research. CW, LC, XL, and QY collected the data and performed the statistical analysis. XW, LC, and QY wrote the first draft of the manuscript. All authors contributed to the article and approved the submitted version.

## Funding

This research was supported by grants from the National Natural Science Foundation of China (32200912), Zhejiang Federation of Humanities and Social Sciences (2023 N013), Young Doctoral Program of Zhejiang Normal University (20214833), Open Research Fund of the Department of Psychology of Zhejiang Normal University, Open Research Fund of College of Teacher Education of Zhejiang Normal University (Nos. jykf22034 and jykf20023), and 2019 Annual Project of Zhejiang Federation of Social Sciences (No. 2019N41).

## Conflict of interest

The authors declare that the research was conducted in the absence of any commercial or financial relationships that could be construed as a potential conflict of interest.

## Publisher’s note

All claims expressed in this article are solely those of the authors and do not necessarily represent those of their affiliated organizations, or those of the publisher, the editors and the reviewers. Any product that may be evaluated in this article, or claim that may be made by its manufacturer, is not guaranteed or endorsed by the publisher.

## Appendix. A version of the questions used in experiments 1 and 2

1. A bat and a ball cost $1.10. The bat costs $1.00 more than the ball. How much does the ball cost?
2. A phone and a headphone cost $1,100. The phone costs $1,000. How much does the headphone cost?
3. If it takes 5 machines 5 min to make 5 widgets, how long would it take 100 machines to make 100 widgets?
4. In a lake, there is no lotus now. Every day, a new lotus blossoms. If it takes 48 days for the lotus to cover the entire lake, how long would it take to cover half of the lake?
5. A frog falls into a 30-meter-deep well. Every day, it climbs 3 m, but at night it falls 2 m. How many days would it take for the frog to climb out of the well?
6. In an examination, Xiao Ming ranked 5th in the class but 47th from the bottom. How many students are there in this class?
7. A phone and a headphone cost $1,100. The phone costs $1,000 more than the headphone. How much does the headphone cost?
8. In a lake, there is a patch of lily pads. Every day, the patch doubles in size. If it takes 48 days for the patch to cover the entire lake, how long would it take for the patch to cover half of the lake?
9. In the Animal Games, the rabbit ranked 3rd. If there are 21 players behind her, how many players are there in the Games?
10. A goldfish and a fish tank cost ¥66. The goldfish cost ¥6. How much does the fish tank cost?
11. If it takes 1 machine 5 min to make 5 widgets, how long would it take 1 machine to make 100 widgets?
12. A frog falls into a 30-meter-deep well. Every day, it climbs 3 m. How many days would it take for the frog to climb out of the well?
13. 400 grams of flower seeds are needed to spread a circular bed 10 meters in diameter. How many grams of flower seeds are required in order to spread a circular bed 20 meters in diameter?
14. A circular flower bed with a diameter of 3 meters needs 180 bricks for its fence. How many bricks are required in order to fence a circular flower bed with a diameter of 6 meters?
15. Ten apple trees can be planted in a square orchard with a side length of 5 meters. How many apple trees can be grown in a square orchard with a side length of 10 meters?
16. It takes a worker 3 days to fence a square yard with a side length of 60 m. How many days does it take to fence a square yard with a side length of 120 m?

Note: There are differences between Chinese and English. The above questions are translated versions. The Chinese version is used in the actual experiments, and the number of characters counted is based on Chinese characters.
